# Cacospongionolide and Scalaradial, Two Marine Sesterterpenoids as Potent Apoptosis-Inducing Factors in Human Carcinoma Cell Lines

**DOI:** 10.1371/journal.pone.0033031

**Published:** 2012-04-03

**Authors:** Daniela De Stefano, Giuseppina Tommonaro, Shoaib Ahmad Malik, Carmine Iodice, Salvatore De Rosa, Maria Chiara Maiuri, Rosa Carnuccio

**Affiliations:** 1 Dipartimento di Farmacologia Sperimentale, Facoltà di Scienze Biotecnologiche, Università degli Studi di Napoli Federico II, Napoli, Italy; 2 National Research Council of Italy-Institute of Biomolecular Chemistry, Pozzuoli (NA), Italy; 3 INSERM U848, Institut Gustave Roussy, Villejuif, France; Maastricht University Medical Center, The Netherlands

## Abstract

Apoptosis, a form of programmed cell death, is a critical defence mechanism against the formation and progression of cancer and acts by eliminating potentially deleterious cells without causing such adverse effects, as inflammatory response and ensuing scar formation. Therefore, targeting apoptotic pathways becomes an intriguing strategy for the development of chemotherapeutic agents. In last decades, marine natural products, such as sesterterpenoids, have played an important role in the discovery and development of new drugs. Interestingly, many of these compounds have a strong potential as anticancer drugs by inhibiting cell proliferation and/or inducing cell death. In the present study, we investigated the effects of scalaradial and cacospongionolide, two sesterterpenoids from *Cacospongia scalaris* and *Fasciospongia cavernosa* marine sponges, on the apoptotic signalling pathway in three different human tumoral cells. Results were obtained by using DNA fragmentation, comet and viability assays, quantification of the mitochondrial transmembrane potential and Western blot. The T47D (human breast carcinoma), A431 (human epidermoid carcinoma), HeLa (human cervix carcinoma) and HCT116 (human colon carcinoma) cells were incubated for 24 h with scalaradial or cacospongionolide. Treatment of T47D cells with scalaradial or cacospongionolide for 24 h brought about a significant increase in DNA migration as well as fragmentation. Moreover, incubation of HCT116 and HeLa cells with scalaradial or cacospongionolide for 24 h caused an increased expression of pro-apoptotic proteins. Furthermore, scalaradial or cacospongionolide, added to HCT116 and HeLa cells overnight, induced a significant and concentration-dependent loss of mitochondrial transmembrane potential, an early apoptosis signalling event. These effects paralleled with those achieved with p50 and p65, NF-κB subunits, nuclear level. In conclusion, scalaradial and cacospongionolide, by determining human cancer cell apoptosis, may represent new promising compounds to inhibit cancer cell proliferation.

## Introduction

Cancer is a leading cause of death in industrialized countries [1]. Although mortality rates have declined in recent years due to earlier detection and more options in treatment, most cancers remain incurable. However, the increase of drug-resistant cancers needs the identification of innovative drugs. It is worthy of note that malignant cells are characterized by deregulated signalling pathways involving proliferation, apoptosis, and angiogenesis [2], [3]. Apoptosis, a form of programmed cell death, is a critical defense mechanism against the formation and progression of cancer and exhibits distinct morphological and biochemical traits [4]. Apoptosis acts by eliminating potentially deleterious cells without causing such adverse effects, as inflammatory response, and ensuing scar formation. Therefore, targeting apoptotic pathways becomes an intriguing strategy for the development of chemotherapeutic agents [4].

In the last decades, marine natural products, and particularly sesterterpenoids, have played an important role in the discovery and development of new drugs for their wide variety of chemical structures and biological activities [5]. The richest marine source of natural products has been soft-bodied organisms that lack physical defences against their predators, and therefore rely on chemical defence mechanisms involving cytotoxic secondary metabolites [6]. Interestingly, many of these compounds have a strong potential as anticancer drugs and their most common mechanisms reported are the inhibition of cell proliferation and/or induction of cell death [4]. It has been demonstrated that scalaradial, extracted from *Cacospongia scalaris*, exerts an anti-inflammatory effect by inhibiting PLA2 activity [7]. Moreover, *in vitro* studies reported that scalaradial inhibited EGF-stimulated Akt [8] as well as NF-κB activation [9]. Cacospongionolide, extracted from *Fasciospongia cavernosa*, is known to inhibit NF-κB activation, leading to an anti-inflammatory effect [10]. Many evidences reported the anti-apoptotic role of NF-κB, which is persistently activated in several malignancies [11].

In the present study, we examined the ability of scalaradial and cacospongionolide to inhibit proliferation and induce apoptosis in several human carcinoma cell lines.

## Results

### Effect of Cacospongionolide and Scalaradial on Cell Viability

**Figure 1:**
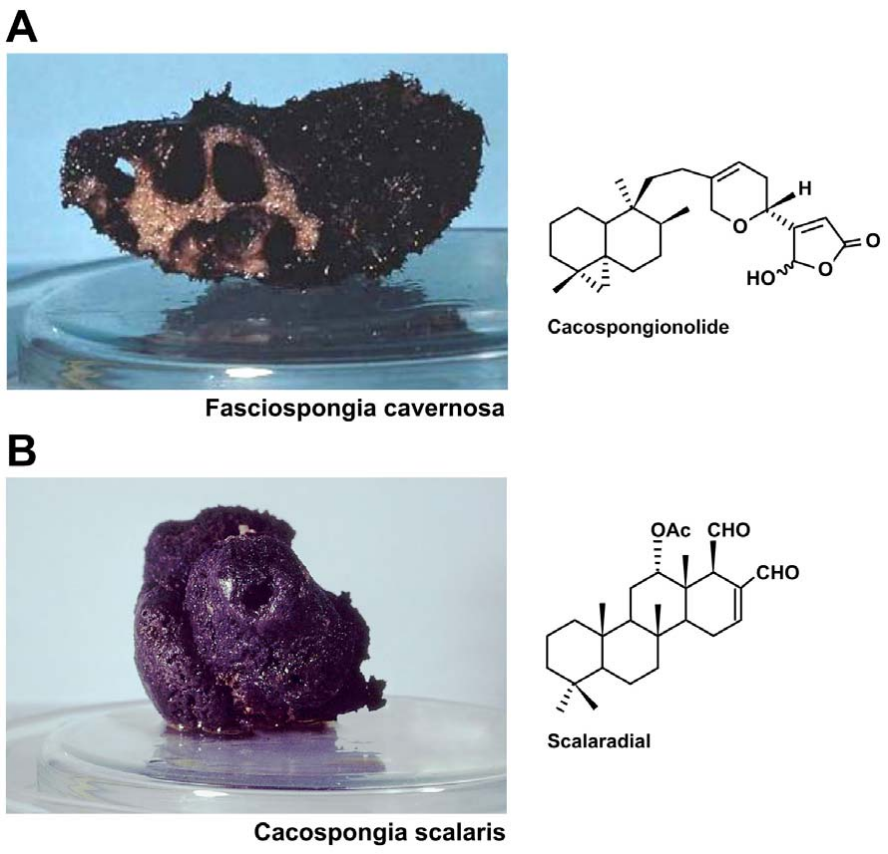
Cacospongionolide and Scalaradial: structures and original sponges. Chemical structures of cacospongionolide and scalaradial isolated from marine sponges *Fasciospongia cavernosa* and *Cacospongia scalaris*, respectively.

**Figure 2:**
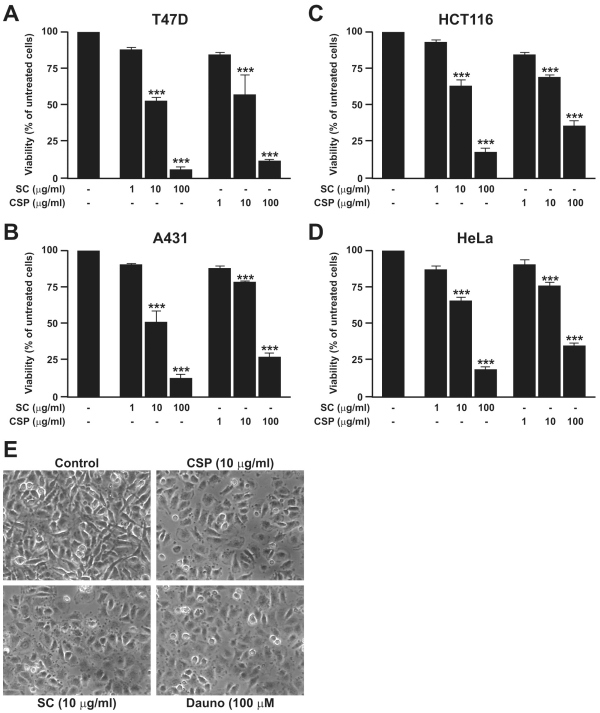
Effect of SC and CSP on cell viability and morphology. T47D (A), A431(B), HCT116(C) and HeLa (D) cells were incubated with SC (1, 10 and 100 µM) and CSP (1, 10 and 100 µM) for 24 h. Thereafter cell viability was determined by MTT assay as described in Materials and Methods. Data are expressed as mean ± S.E.M. of six separate experiments (***p<0.001 *vs.* untreated cells). T47D cells (E) were treated with SC (10 µg/ml), CSP (10 µg/ml) or Dauno (100 µM) for 24 h, visualised by phase-contrast microscopy and photographed. The untreated cells (Control) exhibited normal morphologic aspects, whereas the cells treated with CSP, SC and Dauno were suggestive of apoptosis. Data illustrated in (C) are from a single experiment and are representative of three separate experiments.

The natural compounds scalaradial and cacospongionolide were extracted from the sponges *Cacospongia scalaris* and *Fasciospongia cavernosa*, respectively, as previously reported [12], [13] (Figure 1). Incubation of T47D, A431, HCT116 and HeLa cells with SC (1, 10, and 100 µg/ml) or CSP (1, 10, and 100 µg/ml) for 24 h caused a significant and concentration-dependent reduction of cell viability (Figure 2A-D). The IC50 of SC were 0.024, 0.026, 1.851, 1.284 and 0.52 M for T47D, A431, HCT116, and HeLa , respectively whereas for CSP were 0.030, 0.089, 3.697, 4.302 and 4.49 M. Daunorubicin (100 µM) inhibited in a significant manner cell viability (<20%, data not shown). DMSO did not modify cell viability (<90%, data not shown).

### Effect of SC and CSP on Cell Morphology

We investigated the effect of SC (10 µg/ml) or CSP (10 µg/ml) on the morphologic changes visualised by phase-contrast microscopy of T47D human breast cancer cells after 24 h. The untreated cells showed a normal morphologic aspect. Cells treated with SC or CSP were suggestive of apoptosis by blebs recognition. Dauno exhibited the same effect (Figure 2C).

### Effect of SC and CSP on DNA Fragmentation

**Figure 3:**
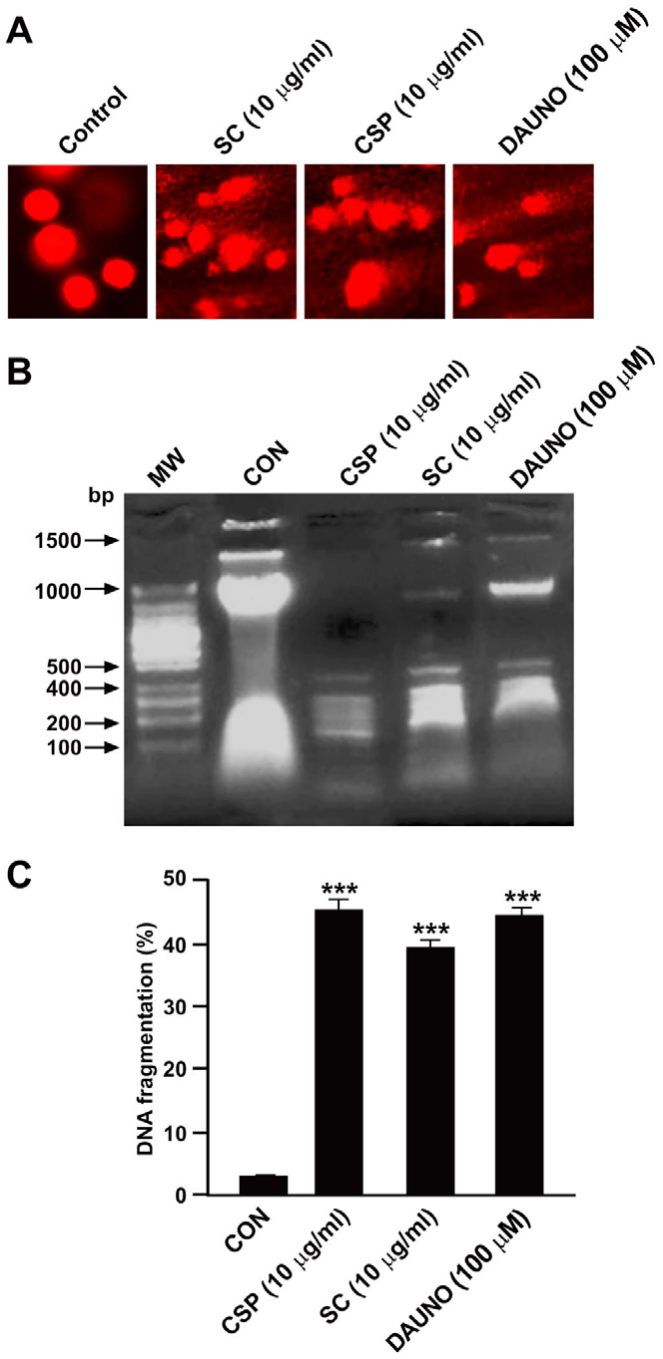
Effect of SC and CSP on DNA fragmentation. The T47D cells were treated with SC (10 µg/ml), CSP (10 µg/ml) or Dauno (100 µM) for 24 h, thereafter DNA fragmentation was analyzed by comet assay (A), agarose gel electrophoresis (B) and diphenylamine assay (C). Basal DNA damage from untreated cells, DNA damage from SC or CSP cells (A). Data are from a single experiment and representative of 50 randomly selected cells stained with ethidium bromide (EtBr). Data illustrated in (B) are from a single experiment and representative of three separate experiments, while data in (C) are expressed as mean ± S.E.M. of three separate experiments. ***p<0.001 *vs.* control (untreated cells).

Internucleosomal DNA degradation determined qualitatively by comet assay (Figure 3A) as well as by agarose gel electrophoresis (Figure 3B) and quantitatively by the diphenylamine reaction (Figure 3C) were analysed as parameters of apoptosis. The T47D cells treated with SC (10 µg/ml), CSP (10 µg/ml) or Dauno (100 µM) for 24 h were used to perform comet assay. Following electrophoresis, the sample is stained with a DNA-binding dye and viewed under a microscope. Short strands of DNA generated from DNA strand breaks and/or alkaline labile sites migrate farther than intact DNA during electrophoresis and form the “tail” of the “comet”. As expected, SC, CSP and Dauno induced a significant increase in DNA migration in human breast carcinoma cells, as compared to control cells (Figure 3A). Moreover, the incubation of T47D cells with SC (10 µg/ml) or CSP (10 µg/ml) for 24 h led to the appearance of oligonucleosomal fragmentation with the characteristic ladder pattern associated with apoptosis and elicited a significant increase of DNA fragmentation (by 45.27±1.8 and 39.48±1.35%, respectively, *n* = 3) as compared to control, the untreated cells (3.12±0.25%). Dauno similarly induced the same effect (44.6±1.25%) (Figure 3B, C).

### Effect of SC and CSP on Apoptotic Signalling Proteins

**Figure 4:**
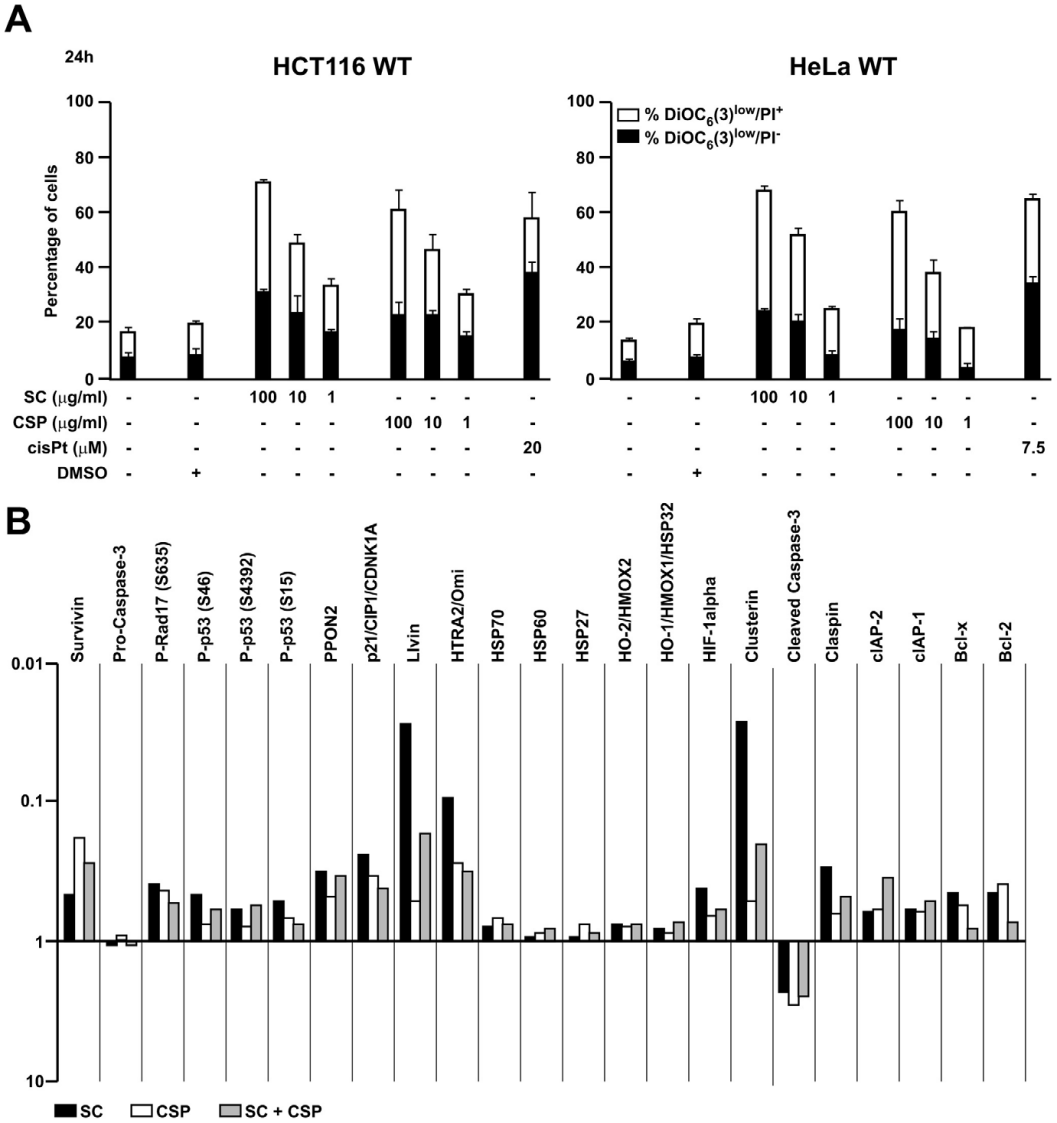
Effect of SC and CSP on apoptosis induction. Detection of dead and dying cells by FACS (A). Cells treated overnight with SC, CSP and cisPt were stained with ΔΨ_m_-sensitive dye DiOC_6_(3) and the vital dye propidium iodide (PI). The white portions of the columns refer to the DiOC_6_(3)^low^/PI^+^ population (dead) and the remaining part of the column corresponds to the DiOC_6_(3)^low^/PI^-^ (dying) population. Results are means ± S.E.M. of three independent experiments. Expression profile of apoptosis-related proteins (B). Cell lysates from HeLa cells, treated with SC (10 µM) and CSP (10 µM) overnight, were analyzed by human apoptosis profiler. Data are expressed as means ± S.D. of two independent experiments.

**Figure 5:**
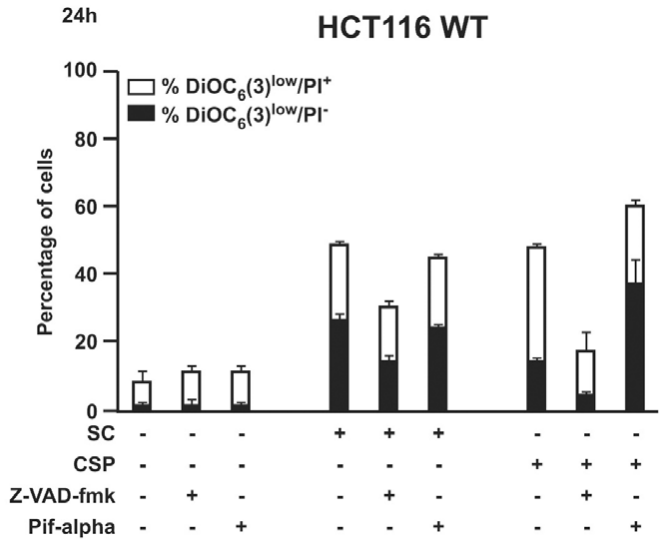
Effect of SC and CSP on cell viability. FACS analysis shows the effect of SC (10 µg/ml) or CSP (10 µg/ml) on HCT116 cell viability in the presence or absence of Z-VAD-fmk (50 µM) and pifthrin-alpha (30 µM). Data are the mean ± SD of three experiments in triplicate.

It has been described that a reduction in mitochondrial transmembrane potential (ΔΨ_m_) precedes apoptosis and may represent an early signalling event [14]. Finally, we evaluated the effect of SC and CSP on the ΔΨ_m_. As shown in Figure 4A, two different human carcinoma cell lines treated overnight with SC and CSP showed a significant and concentration-dependent loss of the mitochondrial transmembrane potential compared to untreated as well as vehicle-treated cells. CisPt exhibited the same effect (Figure 4A). To investigate the involvement of apoptotic signalling molecules following SC and CSP treatment, the expression level of apoptosis related proteins were analyzed by means of an antibody array. For this, HeLa cells were treated with SC (10 µg/ml) or CSP (10 µg/ml) for 6 h. Cell lysates were prepared and apoptosis antibody array was performed following the manufacturer’s protocol. Our results showed that a number of apoptotic signalling proteins were modulated following treatment of SC and CSP. Among the pro-apoptotic proteins present on the array, a high intensity of chemioluminescence was detected for cleaved caspase-3. On the other hand, several anti-apoptotic proteins were significantly inhibited by SC and CSP treatment, such as Survivin, Bcl-2 and IAPs (Figure 4B). Surprisingly, SC and CSP elicited similar changes on the expression level of proteins involved in the apoptosis pathway (Figure 4B). However, often SC showed major effects compared to CSP. Unfortunately, a co-treatment with SC and CSP did not result in an addictive or synergistic effect (data not shown). This kind of epistatic analysis confirms the pro-apoptotic pathways elicited by both agents. In order to deepen our results, we are further investigating the molecular mechanisms at the basis of this expression pattern. Thus, HCT116 cells were incubated with SC or CSP in the presence of the pan-caspase inhibitor Z-VAD-fmk (50 µM) or the p53 inhibitor pifthrin-alpha (30 µM). Results from FACS analysis demonstrated that SC as well as CSP were not capable to cause cell death in the presence of Z-VAD-fmk, thus suggesting that SC and CSP acted *via* caspase (Figure 5). On the contrary, in the presence of pifthrin-alpha SC and CSP caused a significant cell death, suggesting that p53 was not involved (Figure 5). These results suggest that activated caspases are key molecules to determine the effect of SC and CSP.

### Effect of SC and CSP on p50 and p65 Nuclear Translocation

**Figure 6:**
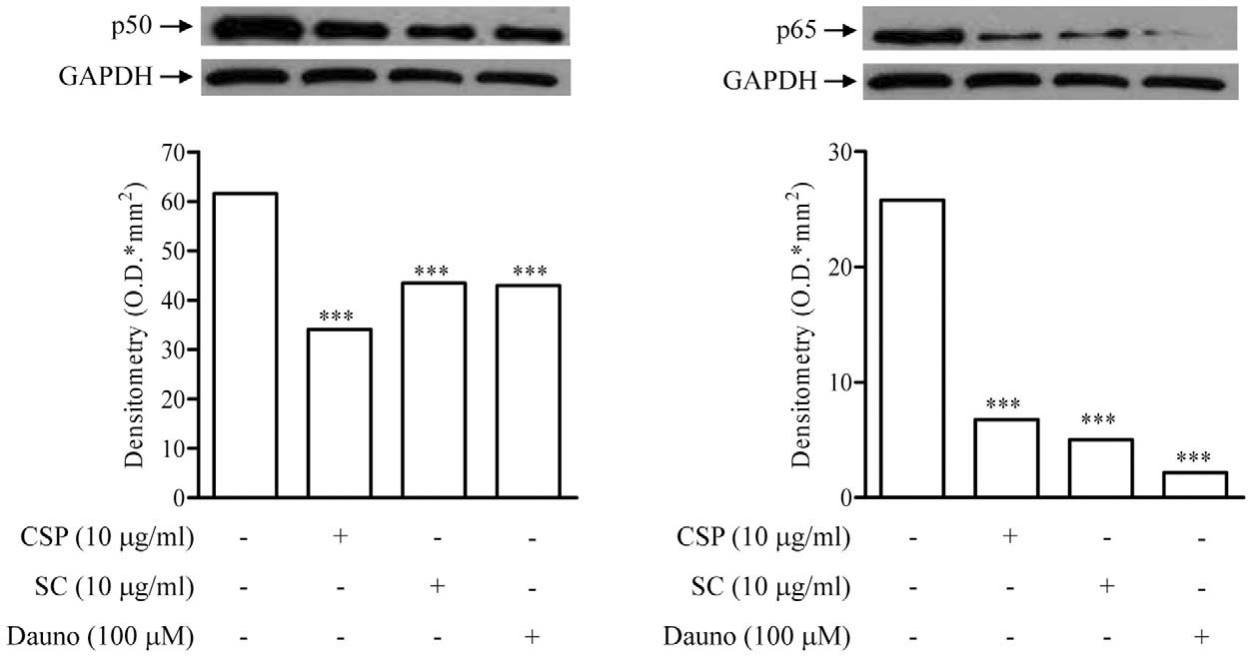
Effect of SC and CSP on p50 and p65 nuclear translocation. Representative Western blot of p50 and p65 as well as the densitometric analysis shows p50 and p65 nuclear translocation in T47D cells incubated with CSP (10 µg/ml) or SC (10 µg/ml) for 24 h. The results are from a single experiment and are representative of three separate experiments. Densitometric data are expressed as mean ± S.E.M. of three separate experiments. ***p<0.001 *vs*. untreated cells. The expression of GAPDH is shown as an equal loading control.

To explore whether the effects of SC and CSP on apoptotic signalling proteins was attributable to NF-κB inhibition, we evaluated T47D nuclear levels of p50 and p65, NF-κB subunits, by Western blot. Treatment of cells with CSP (10 µg/ml) or SC (10 µg/ml) for 24 h reduced nuclear translocation of both p50 (by 44.72±5.73% and 29.45±6.71%, respectively, *n* = 3) and p65 (by 73.77±1.51% and 80.47±0.11%, respectively, *n* = 3). Dauno (100 µM) exhibited the same effects (by 30.29±8.49% and 81.63±0.46%, respectively, *n* = 3) (Figure 6).

## Discussion

Cancer is a malignant disease resulting from several causes [15]. Among these, mutation of oncogenes, and/or tumor suppressor genes can determine the alteration of signalling pathways, including those involved in cancer cell proliferation [16]. One of the most important processes regulating the balance of cell growth and cell death is apoptosis, toward which has been given much attention for cancer treatment [17]. Natural products, including those of marine origin that show cancer-preventive and anticancer properties, attract more attention as probable candidates to be used in cancer preventive or chemotherapeutic strategies [18], [19]. Cacospongionolide has been firstly reported to inhibit PLA2 activity in human neutrophils [12] and, subsequently, to reduce NF-κB transcriptional activity in LPS-stimulated mouse peritoneal macrophages [10]. As for scalaradial, it has been reported to inhibit PLA2 activity as well as EGF-stimulated Akt [7], [8] and NF-κB activation [9]. NF-κB is persistently activated in several malignancies and its anti-apoptotic role has been demonstrated [11]. The results of the present study indicate that scalaradial and cacospongionolide are capable of causing cell death by inducing apoptosis in four different human cancer cells. Preliminary, we found that both scalaradial and cacospongionolide reduced human cancer cell viability in a concentration-dependent manner, as compared to untreated cells. In addition, scalaradial and cacospongionolide induced a type of cell death that bears some hallmarks of apoptosis, such as ΔΨ_m_ dissipation and DNA fragmentation, which were confirmed by optical microscopy, comet assay and FACS. Progresses in cancer biology and genetics have led to the concept that deregulation of apoptosis-related proteins or genes bears prominent effect on the malignant phenotype [20]. In addition, it is widely demonstrated that some oncogenic mutations suppressing apoptosis may lead to tumour initiation, progression or metastasis [21]. Therefore, apoptosis of premalignant or malignant cells would represent a protective mechanism against tumour formation and development. Interestingly, conventional chemotherapy for cancer utilizes cytotoxic agents that elicit their therapeutic effect partly through apoptosis induction, whereas over-expression of anti-apoptotic proteins in cancer cells can inhibit apoptosis and even engender chemoresistance [22]. Furthermore, also radiation therapy is known to induce apoptosis of malignant cells and an inherent resistance is mediated by constitutively activated oncogenic, proliferative and anti-apoptotic proteins/pathways [23]. Therefore, chemotherapeutic interventions often fail to determine complete health in patients. Based on this knowledge, it would be useful to have new drugs overcoming the impact of resistance and increase the anticancer efficacy. Recently, many anti-tumour agents target proteins involved in apoptosis while the effects of these agents might be various, and they either induce cancer cell death or enhance the sensitivity of cancer cells to certain cytotoxic drugs or radiation [24], [25]. Here, we have shown that scalaradial and cacospongionolide exert a significant effect on inhibition of cell growth and induction of apoptosis in four different human tumoral cells by induction of the apoptotic pathway. Particularly, anti-apoptotic proteins were significantly inhibited by scalaradial and cacospongionolide treatment, such as Survivin, Bcl-2 and IAPs. Interestingly, SC or CSP in the presence of the pan-caspase inhibitor Z-VAD-fmk were not capable to cause cell death. In contrast, the p53 inhibitor did not modified the effect of SC and CSP, thus suggesting that SC and CSP did not acted *via* p53. These results suggest that activated caspases are key molecules to determine the effect of SC and CSP. Thus, the significant reduction of several anti-apoptotic proteins expression suggests promising anticancer properties for both compounds. It is well known that many pro- and anti-apoptotic proteins are regulated at transcriptional level by NF-κB [26]. Here, we reported that scalaradial and cacospongionolide were capable of inhibiting p50 and p65 nuclear translocation. However, further investigations are necessary for a more detailed understanding of the molecular mechanisms by which cacospongionolide and scalaradial induce cancer cell apoptosis. In conclusion scalaradial and cacospongionolide, by determining human cancer cell apoptosis, may represent a new promising compounds to inhibit cancer cell proliferation.

## Materials and Methods

### Ethics Statement

No specific permits were required for the described field studies. No specific permissions were required for collecting these sponges because the location they were taken from is not privately-owned or protected in any way. In addition, this study does not involve endangered or protected species.

### Isolation of Scalaradial and Cacospongionolide

Scalaradial (SC) was isolated from the sponge *Cacospongia scalaris*, while Cacospongionolide (CSP) was isolated from the sponge *Fasciospongia cavernosa*. Both sponges were collected in Rovijni (Croatia) in summer 2007, and kept in freezer at -20°C until analysis. The isolation of SC and CSP was performed as previously described [12], [13]–[26].

### Cell Culture

T47D (breast carcinoma), A431 (epidermoid carcinoma), HeLa (cervix adenocarcinoma) human cells were cultured in Dulbecco’s modified Eagle’s medium (DMEM) containing 10% fetal calf serum (FCS), 1 mM pyruvate and 10 mM Hepes at 37°C under 5% CO_2_. HCT116 human colon carcinoma cells were cultured in McCoy’s 5A medium containing 10% FCS, 100 mg/l sodium pyruvate, 10 mM HEPES buffer, 100 U/ml penicillin G sodium, and 100 µg/ml streptomycin sulfate (37°C, 5% CO_2_). All media and supplements for cell culture were purchased from Gibco-Invitrogen (Carlsbad, USA). Cells (3×10^5^) were seeded in 24-well plates and grown for 24 h before treatment with scalaradial (SC at a final concentration/per well 1, 10 and 100 µg/ml, which is 0.0026, 0.026 and 0.26 M, respectively), cacospongionolide (CSP at a final concentration/per well 1, 10 and 100 µg/ml which is 0.0025, 0.025 and 0.25 M, respectively), daunorubicin (Dauno at a final concentration/per well 100 µM) or cisPlatinum (cisPt at a final concentration/per well 7.5, 15, 20 and 40 µM) for the indicated period. Cell lines were all from ATCC catalogue. In some experiments, cells were incubated with the pan-caspase inhibitor Z-VAD-fmk (50 µM) or the p53 inhibitor pifthrin-alpha (30 µM) in the presence of SC or CSP.

### MTT Viability Assay

The cells were plated in 96 culture wells at a density of 250.000 cells/ml and allowed to adhere for 2 h. Thereafter the medium was replaced with fresh medium and cells were incubated with SC, CSP or Dauno alone or in association. After 24 h, cell viability was determined by using 3-(4,5-dimethylthiazol-2yl)-2,5-diphenyl-2H-tetrazolium bromide (MTT) conversion assay [27]. Briefly, 125 µl of MTT (5 mg/ml in complete DMEM) were added to the cells and incubated for additional 3 h. After this time point the cells were lysed and the dark blue crystals solubilized with 375 µl of a solution containing 50% (v:v) N,N-dymethylformamide, 20% (w:v) SDS with an adjusted pH of 4.5. The optical density (OD) of each well was measured with a microplate spectrophotometer (Titertek Multiskan MCCC/340) equipped with a 620 nm filter. The cell viability in response to treatment with test compounds was calculated as % cell viability = (OD treated/OD control)×100.

### Morphological Investigation

T47D cells (1×10^6^ cells/ml) were plated in 35×10 mm diameter culture dishes and incubated with SC (10 µg/ml), CSP (10 µg/ml) or Dauno for 24 h. Thereafter, the cells were washed three times with PBS, fixed with 3% formaldehyde in PBS, and then visualised by phase-contrast microscopy (12,5x; LAS, Leica).

### Comet Assay

The comet assay was performed according to a standard protocol [28]. Aliquots of 5 µl cell suspension (about 15000 T47D cells treated as described above) were mixed with 120 µl low melting point agarose (0.5% in phosphate-buffered saline) and added to microscope slides, which had been covered with a bottom layer of 1.5% agarose. Slides were lysed (pH 10; 4°C) for at least 1 h and processed using a time of alkali denaturation of 25 min and electrophoresis (0.86 V/cm) of 25 min at a pH>13. Slides were washed three times in Tris/EDTA (TE) buffer (100 µM Tris, 5 µM EDTA, pH 10). After removing the coverslips, slides were processed as usual. Slides were coded and images of 50 randomly selected cells stained with ethidium bromide (EtBr) were analysed from each slide and visualized by fluorescence microscopy (5x; LAS, Leica).

### Gel electrophoresis of Fragmented DNA

T47D (2×10^6^) cells were seeded on 10 cm diameter Petri dishes and allowed to adhere for 2 h. Thereafter the medium was replaced with fresh medium and the cells were incubated as described above for 24 h. For analysis of genomic DNA, cells were gently scraped off and collected together with non-attached cells in the supernatant. Cells were resuspended in 250 µl of Tris-Borate EDTA (TBE) buffer containing 0.25% NP-40 and 100 µg/ml RNAse A. After incubation at 37°C for 30 min., extracts were treated with 1mg/ml proteinase K for additional 30 min. at 37°C. Then equal amounts (10 µl) of the extracts were loaded on a 1% agarose gel containing (1 µg/ml) ethidium bromide and run for 1h in 1×TBE at 90 V. The gel captions were obtained with Polaroid T667.

### Quantification of DNA Fragmentation

DNA fragmentation was performed essentially as previously described by Messmer and Brune [29] with some modification. Briefly, T47D cells (4×10^6^) were seeded on 10 cm diameter culture dishes and incubated with CSP, SC or Dauno for 24 h. Thereafter, the cells were scraped off the culture plates, centrifuged at 180×g for 10 min, resuspended in 250 µl of TE-buffer (10 mM Tris, 1 mM EDTA, pH 8.00), and incubated with an additional volume of lysis buffer (5 mM Tris, 1 mM EDTA, pH 8.00, 0.5% Triton X-100) for 30 min at 4°C. After lysis, the intact chromatin (pellet) was separated from DNA fragments (supernatant) by centrifugation for 15 min at 13 000 rpm. Pellet was resuspended in 500 µl of TE-buffer and samples were precipitated by adding 500 µl of 25% trichloroacetic acid at 4°C. Samples were pelleted at 3000×g for 10 min and the supernatant was removed. After addition of 300 µl of 5% trichloroacetic acid, samples were boiled for 15 min. DNA contents were quantified by using the diphenylamine (DPA) reagent (0.3 g DPA, 0.2 ml of sulphuric acid, 0.1 ml of 1.6% acetaldehyde, 10 ml of acetic acid glacial). The percentage of fragmented DNA was calculated as the ratio of the DNA content in the supernatant to the amount in the pellet.

### Flow Cytometry

The following fluorochromes were employed to determine apoptosis-associated changes by cytofluorometry: 3,3′-dihexyloxacarbocyanine iodide (DiOC_6_(3), 40 nM) for quantification of the mitochondrial transmembrane potential (ΔΨ_m_) and propidium iodide (PI; 1 µg/ml) (both from Molecular probes) for determination of cell viability [30]. HeLa and HCT116 cells were trypsinized and labeled with the fluorochromes at 37°C, followed by cytofluorometric analysis with a fluorescence-activated cell sorter (FACS) scan (Becton Dickinson).

### Apoptosis Array

The expression profile of apoptosis-related proteins was detected and analyzed using a human apoptosis array kit (ARY009), according with the manufacturer’s instructions (R&D Systems). Briefly, the membrane containing immobilized apoptosis-related antibodies was blocked with bovine serum albumin for 1 h on a rocking platform at room temperature. The membrane was then incubated with lysates of untreated or treated HeLa cells (for 6h with SC, 10 µg/ml, or CSP, 10 µg/ml) along with Detection Antibody Cocktail overnight at 2°C to 8°C on a rocking platform. The membrane was incubated with streptavidin-horseradish peroxidase conjugate followed by chemiluminescent detection reagent. The membrane was scanned and pixel density was presented by quantifying the mean spot densities from two separate experiments. For quantification, the spot volume was determined, corrected for background and expressed as fold change (treated *vs.* untreated cells).

### Western Blot Analysis

Cytosolic and nuclear fraction proteins from T47D cells incubated for 24 h as described above were prepared as previously described [27] and mixed with gel loading buffer (50 mM Tris, 10% SDS, 10% glycerol, 10% 2-mercaptoethanol, 2 mg/ml of bromophenol) in a ratio of 1:1, boiled for 3 min and centrifuged at 10,000 g for 5 min. Protein concentration was determined and equivalent amounts (30 µg) of each sample were electrophoresed in a 8–12% discontinuous polyacrylamide minigel. The proteins were transferred onto nitro-cellulose membranes, according to the manufacturer’s instructions (Protran, Schleicher & Schuell, Dassel, Germany). The membranes were saturated by incubation at room temperature for 2h with 10% non-fat dry milk in phosphate buffer saline (PBS) and then incubated with (1:1000) anti-p50 or anti-p65 at 4°C overnight. The membranes were washed three times with 0.1% Tween 20 in PBS and then incubated with anti-rabbit, anti-mouse or anti-goat immunoglobulins coupled to peroxidase (1:1000) (DAKO, Milan, Italy). The immunocomplexes were visualised by the ECL chemiluminescence method (Amersham, Milan, Italy). The membranes were stripped and re-probed with GAPDH antibody to verify equal loading of proteins. Subsequently, the relative expression of p50 and p65 proteins in nuclear fractions was quantified by densitometric scanning of the X-ray films with a GS 700 Imaging Densitometer and a computer programme (Molecular Analyst, IBM).

### Statistical Analysis

Results are expressed as the means ± SEM of *n* experiments. Statistical significance was calculated by one-way analysis of variance (ANOVA) and Bonferroni-corrected P-value for multiple comparison test. The level of statistically significant difference was defined as p<0.05.

## References

[1] World Health Organization (2005). Global action against cancer..

[2] Al-Ejeh F, Kumar R, Wiegmans A, Lakhani SR, Brown MP (2010). Harnessing the complexity of DNA-damage response pathways to improve cancer treatment outcomes.. Oncogene.

[3] Udagawa T, Wood M (2010). Tumor-stromal cell interactions and opportunities for therapeutic intervention.. Curr Opin Pharmacol.

[4] Lin X, Liu M, Hu C, Liao DJ (2010). Targeting cellular proapoptotic molecules for developing anticancer agents from marine sources.. Curr Drug Targets.

[5] De Rosa S, Mitova M (2005). “Bioactive marine sesterterpenoids” in Studies in Natural Products Chemistry, Ed. Atta-ur-Rahman, Elsevier, Amsterdam, Netherlands Vol.. 32,.

[6] Napolitano JG, Daranas AH, Norte M, Fernández JJ (2009). Marine macrolides, a promising source of antitumor compounds.. Anticancer Agents Med Chem.

[7] Liu Y, Levy R (1997). Phospholipase A2 has a role in proliferation but not in differentiation of HL-60 cells.. Biochim Biophys Acta.

[8] Xie Y, Liu L, Huang X, Guo Y, Lou L (2005). Scalaradial inhibition of epidermal growth factor receptor-mediated Akt phosphorylation is independent of secretory phospholipase A2.. J Pharmacol Exp Ther.

[9] Ruipérez V, Casas J, Balboa MA, Balsinde J (2007). Group V phospholipase A2-derived lysophosphatidylcholine mediates cyclooxygenase-2 induction in lipopolysaccharide-stimulated macrophages.. J Immunol.

[10] Posadas I, De Rosa S, Terencio MC, Payá M, Alcaraz MJ (2003). Cacospongionolide B suppresses the expression of inflammatory enzymes and tumour necrosis factor-alpha by inhibiting nuclear factor-kappa B activation.. Br J Pharmacol.

[11] Karin M (2009). NF-kappaB as a critical link between inflammation and cancer.. Cold Spring Harb Perspect Biol.

[12] De Rosa S, De Stefano S, Zavodnik N (1988). “Cacospongionolide: a new antitumoral sesterterpene, from the marine sponge *Cacospongia mollior*”.. J Org Chem.

[13] De Rosa S, Crispino A, De Giulio A, Iodice C, Pronzato R (1995). “Cacospongionolide B, a new sesterterpene, from the marine sponge *Fasciospongia cavernosa*” J Nat Prod.

[14] Zamzami N, Marchetti P, Castedo M, Decaudin D, Macho A (1995). Sequential reduction of mitochondrial transmembrane potential and generation of reactive oxygen species in early programmed cell death.. J Exp Med.

[15] Rook GA, Dalgleish A (2011). Infection, immunoregulation, and cancer.. Immunol Rev.

[16] Chaturvedi MM, Sung B, Yadav VR, Kannappan R, Aggarwal BB (2011). NF-κB addiction and its role in cancer: ‘one size does not fit all’.. Oncogene.

[17] Beth L (2007). Autophagy and cancer.. Nature.

[18] Fedorov SN, Radchenko OS, Shubina LK, Balaneva NN, Bode AM (2006). Evaluation of cancer-preventive activity and structure-activity relationships of 3-demethylubiquinone Q2, isolated from the ascidian *Aplidium glabrum*, and its synthetic analogs.. Pharm Res.

[19] Fedorov SN, Shubina LK, Bode AM, Stonik VA, Dong Z (2007). Dactylone inhibits epidermal growth factor-induced transformation and phenotype expression of human cancer cells and induces G1-S arrest and apoptosis.. Cancer Res.

[20] Liu JJ, Lin M, Yu JY, Liu B, Bao JK (2011). Targeting apoptotic and autophagic pathways for cancer therapeutics.. Cancer Lett.

[21] Demaria S, Pikarsky E, Karin M, Coussens LM, Chen YC (2010). Cancer and inflammation: promise for biologic therapy.. J Immunother.

[22] Richardson A, Kaye SB (2008). Pharmacological inhibition of the Bcl-2 family of apoptosis regulators as cancer therapy.. Curr Mol Pharmacol.

[23] Deorukhkar A, Krishnan S (2010). Targeting inflammatory pathways for tumor radiosensitization.. Biochem Pharmacol.

[24] De Rosa S, Crispino A, De Giulio A, Iodice C, Benrezzouk R (1998). A new cacospongionolide inhibitor of human secretory phospholipase A2 from the Tyrrhenian sponge *Fasciospongia cavernosa* and absolute configuration of cacospongionolides.. J Nat Prod.

[25] Rueda A, Zubia E, Ortega MJ, Carballo JL, Salva J (1997). New cytotoxic metabolites from the sponge *Cacospongia scalaris* J Org Chem.

[26] Gyrd-Hansen M, Meier PIAPs:fromcaspaseinhibitorstomodulatorsofNF-kappaB, inflammation, cancer (2010). Nat Rev Cancer.

[27] De Stefano D, Ungaro F, Giovino C, Polimeno A, Quaglia F (2011). Sustained inhibition of IL-6 and IL-8 expression by ODN decoy to NF-κB delivered through respirable large porous particles in LPS-stimulated cystic fibrosis bronchial cells.. J Gene Med.

[28] Balasubramanyam M, Adaikalakoteswari A, Sameermahmood Z, Mohan V (2010). Biomarkers of oxidative stress: methods and measures of oxidative DNA damage (COMET assay) and telomere shortening.. Methods Mol Biol.

[29] Messmer UK, Brüne B (1997). Attenuation of p53 expression and Bax down-regulation during phorbol ester mediated inhibition of apoptosis.. Br J Pharmacol.

[30] Maiuri MC, Le Toumelin G, Criollo A, Rain JC, Gautier F (2007). Functional and physical interaction between Bcl-X(L) and a BH3-like domain in Beclin-1.. EMBO J.

